# Morphogens, modeling and patterning the neural tube: an interview with James Briscoe

**DOI:** 10.1186/s12915-014-0105-1

**Published:** 2015-01-20

**Authors:** James Briscoe

**Affiliations:** NIMR, London, NW7 1AA UK

## Abstract

James Briscoe has a BSc in Microbiology and Virology (from the University of Warwick, UK) and a PhD in Molecular and Cellular Biology (from the Imperial Cancer Research Fund, London, now Cancer Research UK). He started working on the development of the neural tube in the lab of Tom Jessel as a postdoctoral fellow, establishing that there was graded sonic hedgehog signaling in the ventral neural tube. He is currently a group leader and Head of Division in Developmental Biology at the MRC National Institute for Medical Research (which will become part of the Francis Crick Institute in April 2015). He is working to understand the molecular and cellular mechanisms of graded signaling in the vertebrate neural tube.

We interviewed him about the development of ideas on morphogenetic gradients and his own work on modeling the development of the neural tube for our series on modeling in biology.

## How did the idea of morphogens that control the patterning of early embryonic tissues first arise?

Trying to understand embryonic patterning has fascinated biologists for more than a century and the idea that a morphogen, that is a graded signal, might control pattern formation (the arrangement of cell type differentiation in tissues) has been around for about as long. The term ‘morphogen’ was actually coined in the 1950s by Alan Turing, but I think the morphogen concept, as we currently understand it, really arose from the work of Lewis Wolpert in the 1960s. He described what we now consider to be a morphogen: the idea that a graded signal, a protein or small molecule signal emanating from a localized source within a tissue, spreads from that source to form a gradient and then cells within that tissue respond to that gradient to adopt different cell identities depending on the amount of the signal that they receive.

## Your recent work has challenged and refined a general theory - the affinity-threshold model of a morphogen. Could you explain how you think that works?

Yes, but I must emphasize that we are certainly not the first to propose or challenge an affinity-threshold model of morphogen interpretation. The affinity-threshold model really emerged from the work of Wolfgang Driever and Christiane Nüsslein-Volhard in the mid to late 1980s. As you may know, this was a period of great excitement in *Drosophila* genetics, when Christiane Nüsslein-Volhard and Eric Wieschaus had just finished their amazing genetic screen, which identified many of the genes that are important in early development. Wolfgang Driever working with Christiane Nüsslein-Volhard was trying to figure out how one of these genes works, a gene called *Bicoid. Bicoid* turns out to encode a transcription factor that is actually distributed in a gradient in the early embryo and some really elegant experiments suggested that different concentrations of Bicoid were responsible for defining different regions of the early *Drosophila* embryo. In a classic paper in 1989 Driever and colleagues provided evidence that Bicoid could activate the expression of these genes and that higher affinity binding sites in the promoter of a gene extended its activation to lower levels of Bicoid [1].

In this model, because a high-affinity binding site only needs a low amount of the transcription factor to activate gene expression it activates at long range, whereas a gene associated with a low-affinity binding site needs a lot of the transcriptional activator for its activation. This became a central idea - that there is a correlation between the affinity and range of activation of a gene.

But it turns out that is an oversimplification. Although it is still a prevalent idea in developmental biology, it has been challenged now for 10 years or more - for example, a really nice paper from Steve Small’s lab from 2005 challenged the idea for Bicoid [2].

In the neural tube, the system that I am interested in, our experiments and the studies of others also lead to a similar conclusion - that the affinity of binding sites that are morphogen responsive is not sufficient to explain how genes respond [3].

## What is the morphogen that acts in patterning of the neural tube, and what were the events you were looking at?

I am really focused on the spinal cord, which is the posterior part of the embryonic neural tube. Like the rest of the central nervous system the spinal cord contains different types of neurons located at different positions along the dorsal-ventral axis. The ventral region of the spinal cord is closest to your belly and the dorsal part is closest to the skin of your back, and along this dorsal-ventral axis different types of neurons are generated during embryogenesis. For example, the motor neurons, which project to muscles, are located and generated in the ventral neural tube. We are interested in how that organization emerges and is elaborated on during early embryogenesis. The key signaling molecule for the ventral neural tube is a member of the Hedgehog family of secreted glycoproteins, called Sonic hedgehog (Shh). Shh is secreted by groups of cells at the ventral pole, so ventral to the neural tube, and it forms a gradient from ventral to dorsal within the neural tube. This gradient is responsible for setting up the pattern of different neurons generated. My work for the last 15 or more years has focused on understanding how Hedgehog does this in the neural tube.

## So if Sonic hedgehog provides the morphogen gradient, what about the response of the cells, and the affinity-threshold idea?

The work in my lab over the last few years has shown that in cells responding to Shh in the neural tube, a series of transcription factors form a network of regulatory interactions. Experimental work - for example, where we knock out one or more of the genes encoding these factors in mice - has revealed that the regulatory interactions between transcription factors are really important to establish the pattern of neurogenesis. So we were interested in trying to understand how the combination of the graded signal from Hedgehog together with the regulatory network establish the pattern of gene expression, which in turn directs the pattern of neurogenesis. It was this motivation that really got us thinking about how you combine the gradient and the affinity for a morphogen effector with a transcriptional network. And there was also one additional complication in the case of the Hedgehog signaling pathway. Unlike some other signaling pathways, Hedgehog does not simply activate a latent activator, but instead converts a transcription factor that represses expression to one that activates gene expression. This adds another dimension, another level of complexity. We were interested in trying to combine these observations to really understand how they give such a well-defined spatial pattern of gene expression in the neural tube.

## And this is what led you to resort to modeling?

I come from an experimental biology background but it became pretty obvious to me 6 or 7 years ago that the type of data we were generating and the complexity of the mechanism meant that it would be very difficult to continue to interpret the data using the usual ways us biologists do it - qualitatively evaluating what is going on. So we made a very determined effort to introduce modeling as a way of assimilating the data, and as a tool to allow us to interpret the data we are generating. This was the start of a continuing collaboration with Karen Page in the Mathematics Department at UCL. One of the difficulties in trying to understand how a morphogen interacts with the transcriptional network is to understand the transcriptional network itself. The transcriptional network of the neural tube is no exception. Transcriptional networks tend to have lots of feedback in them and it is very difficult to understand how a network with feedback operates. It rapidly becomes difficult to follow all the possibilities without a formal model: a mechanistic model to keep track of the details. It was really this motivation that got us into modeling and has led us to try and use modeling side by side with the experimental work to help us interpret data and to develop ideas about new experiments.

## This is the work you describe in a very recent paper in *Development*?

Yes. What we were trying to ask in that particular paper [3] was how the gradient morphogen input from Hedgehog signaling combined with the transcriptional network could give you the spatial patterns of gene expression that generate the pattern of neurogenesis. We wanted to understand the regulatory logic or the topology of the network, to see if it explained the spatial and temporal pattern of gene expression we were seeing in the neural tube. We knew a lot already, we knew from genetic experiments the topology of the networks - the regulatory relationships between reverse transcription factors - and some really nice bioinformatics work from the lab of Johan Ericson about a year or so ago identified the potential binding affinities for the morphogen effector, the Hedgehog effector, the Gli proteins associated with the transcription factors [4].

So that was our starting point, and we wanted to ask with that information could we build a dynamic systems model, a mathematical model of the network that would explain the spatial pattern of gene expression. The particular mathematical formulation we used actually comes from statistical thermodynamics and was introduced to describe gene regulation in the mid-1980s [5]. It is probably less familiar to people than Hill functions, which are generally used to describe gene regulation, but one of the advantages we found using this formulation is that it explicitly describes binding affinity of transcription factors and also has a very simple description for when you have several inputs integrated into the regulation of the gene. This was exactly our assumption: that each of the genes in the network would have activity dependent on up to three distinct inputs. One from the morphogen Hedgehog signal itself, one from broadly and uniformly expressed transcriptional activators - you can think of those as the neural competence transcription factors - and then a third input from the regulatory network itself, so cross-regulation within the network.

We used this formulism to describe that network. Then with a colleague, Chris Barnes in the Cell and Developmental Biology Department at UCL, we used a parameter search technique, called approximate Bayesian computation, which is a powerful computational method to find parameters that allow the model to perform in a way we required. So it is that combination of describing gene regulation using this formulation and that parameter search technique that led us to a mathematical model combing the described differences in binding affinity for the Hedgehog morphogen effectors and the gene regulatory network.

## This sounds very computational - did experimental data play a part in motivating formulation of this model?

The model in the *Development* paper was motivated by experimental data but that particular paper was entirely computational/theoretical. But this was one of a series of papers that we have published over the last 2 or 3 years and in other papers we have combined theoretical/modeling work and experimental data. For example, the paper in 2012 by Balaskas *et al.* [6] describes the transcriptional network on the basis of experimental data, which then led to a mathematical model. Analysis of this model *in silico* resulted in predictions that we could test experimentally. One of the interesting predictions was that the level of signaling necessary to induce a particular responding gene is higher than is necessary for the maintenance of that gene once induced. This is like a memory effect in that once the system has seen the signal it is easier to maintain a particular gene expression state. That prediction came out of the mathematical model but we were then able to go back and experimentally test this [6]. It was an example for us as to how the modeling really helped refine our thinking and also explained an observation that had been bugging us for some time: how are patterns of gene expression maintained in tissues even when signals are changing over time.

## Returning to our initial discussions of our understanding of patterning in early development, do you think the concept of a morphogen gradient is still valid in biology?

That is a good question! I think concepts are useful when they summarize a set of common mechanisms or strategies and the concept of a morphogen gradient, as we discussed, has been around for a while so I am certainly not about to throw it away. But I think we need to refine our ideas. When we look at different tissues that appear to be patterned by morphogens, whether the vertebrate neural tube or the *Drosophila* embryo, we now know a lot of the molecular detail and this is clearly different for different tissues. Nevertheless, can we start to identify design principles that go beyond such details and define common features, even though the details are going to differ between different tissues? Judging from the comparison between Bicoid in the early *Drosophila* embryo and Shh in the neural tube, I think that is going to be the case and it will be exciting over the next few years to see that more fundamental level of similarity.

## How do you think recently developed technologies may help with this?

It is the introduction of new technologies that often drives new understanding in biology and there are two directions where technology is really helping and that excite me. One is high resolution quantitative imaging: the ability to follow at a single cell resolution in a living tissue the activity of individual genes and signaling pathways. This is now enabling us to collect more quantitative data to allow us to build more detailed quantitative models and to test some of the predictions we already have.

The other direction is the genomics. We can go beyond individual genes and look at the entire genome level at how transcriptional networks are controlling cell states and gene activity within individual cells. One of the challenges in the future will be to bring these two types of analyses together: the very detailed quantitative information about individual responding genes/signaling pathways with the 'big data', the whole genomic level data, from RNA sequencing and chromatin interpretation-type experiments. I think this is where we are likely to find new questions and insights into these processes.

## To go back to the neural tube, all the time the pattern is forming, the tissue is also growing - how do you incorporate that growth in your model?

We have used some mathematical tools to model growth in the neural tube. The motivation for modeling in this work was to allow us to assimilate our data: the question there is whether the data explain what we think they explain. In that case the first author, Anna Kicheva, did very detailed painstaking quantitative measurements of the different aspects of neural tube growth. The neural tube grows quite considerably over the period of time when pattern is established and, to see the role of growth in the elaboration of this pattern, she measured parameters such as the rate of proliferation of cells in different regions of the neural tube and the rate at which the cells differentiated into postmitotic neurons.

We collected a lot of quantitative data but then to really understand what it meant we needed a model. So the model is a formal description of growth: growth is a combination of the proliferation that increases cell number and differentiation that will decrease cell number. So if we can measure each of these numbers over time, do they make sense when we look at the overall growth of the neural tube?

By combining this information from wild-type mice, a strain of *Minute* mice (which are smaller than normal) and chick embryos, she could use that quantitative data to identify two distinct phases in neural tube development. An early phase where graded early signals (for example, Hh signaling) are established and patterned, and a second phase where such early established patterning is elaborated by the different rate of growth of the different regions of the neural tube [7]. One interesting side observation is that the different rates of growth are not due to changes in proliferation rate but due to differences in differentiation rate at different regions of the neural tube.

This is a different use of mathematical tools to the models of the transcriptional network we discussed initially. In the case of looking at growth, we are measuring parameters directly rather than trying to infer the parameters from other experiments so it is much more a straightforward, quantitative approach of taking as accurate measurements as possible of the relevant parameters and then test whether the data make sense when we combine all of the measurements together.

## What more would you like to use mathematical modeling for to advance our understanding of patterning in the neural tube?

What I would say is that I don’t see mathematical modeling as separate from the rest of what we do. We see it as an integrated part of our approach now, so it’s really just one of the tools, one of the set of techniques and approaches that we are using, so an alternative way of answering that question is that ultimately we want to be able to describe the development of the neural tube. I don’t expect that to be a single model, it is going to be several different models that together would be able to accurately describe and predict neural tube growth.

## Are there any limitations to what can be achieved by modeling?

Yes, any model is just a description of what you are thinking in formal mathematical terms (there is no magic!). But rigorously describing what you are thinking often has a real advantage. What mathematical modeling does, and I think Jeremy makes this point nicely in his piece [8], is to force you to write down your assumptions. It forces you to describe accurately what you are thinking. So there are no restrictions in those terms, it is more about how you use it and, like any other aspect of biology, your own imagination and creativity.

## What advice would you give other groups who are thinking of embarking on using such collaborations and tools with their experimental data?

My experience is that it has been challenging but very fulfilling. It takes longer than I anticipated to get to a point with collaborations where you are talking the same language and they are productive. I think that is something that you don’t really appreciate until you start these collaborations. Building these relationships over a long term really pays off because initially you spend a lot of time talking past each other. It takes quite a while for one another to understand what each other are trying to achieve, to really understand what can be done and what are the limitations, both of the mathematical modeling and of the experimental approaches. And I think for us as experimental biologists, gaining a sufficient understanding of the modeling, the mathematical techniques, is important in order to be able to really talk to our collaborators. It is challenging, but definitely achievable. I actually think it is easier to learn the maths than it is for the mathematicians to learn the biology - there is just so much biology but not so many mathematical techniques you’re likely to need.

## Note

This article is part of an article collection in *BMC Biology: Beyond Mendel: modeling in biology*. Other articles in this series can be found at [9].
